# Rural outreach by specialist doctors in Australia: a national cross-sectional study of supply and distribution

**DOI:** 10.1186/1478-4491-12-50

**Published:** 2014-09-04

**Authors:** Belinda G O’Sullivan, Catherine M Joyce, Matthew R McGrail

**Affiliations:** School of Rural Health, Office of Research, Monash University, Level 3, 26 Mercy St, PO Box 666, Bendigo, Victoria 3550 Australia; Department of Epidemiology and Preventive Medicine, The Alfred Centre, Monash University, Level 6, 99 Commercial Road, Melbourne, Victoria 3004 Australia; School of Rural Health, Monash University, Northways Road, Churchill, Victoria 3842 Australia

**Keywords:** rural, remote, outreach, visiting, medical, workforce, hub, service planning, policy

## Abstract

**Background:**

Outreach has been endorsed as an important global strategy to promote universal access to health care but it depends on health workers who are willing to travel. In Australia, rural outreach is commonly provided by specialist doctors who periodically visit the same community over time. However information about the level of participation and the distribution of these services nationally is limited. This paper outlines the proportion of Australian specialist doctors who participate in rural outreach, describes their characteristics and assesses how these characteristics influence remote outreach provision.

**Methods:**

We used data from the Medicine in Australia: Balancing Employment and Life (MABEL) survey, collected between June and November 2008. Weighted logistic regression analyses examined the effect of covariates: sex, age, specialist residential location, rural background, practice arrangements and specialist group on rural outreach. A separate logistic regression analysis studied the effect of covariates on remote outreach compared with other rural outreach.

**Results:**

Of 4,596 specialist doctors, 19% (n = 909) provided outreach; of which, 16% (n = 149) provided remote outreach. Most (75%) outreach providers were metropolitan specialists. In multivariate analysis, outreach was associated with being male (OR 1.38, 1.12 to 1.69), having a rural residence (both inner regional: OR 2.07, 1.68 to 2.54; and outer regional/remote: OR 3.40, 2.38 to 4.87) and working in private consulting rooms (OR 1.24, 1.01 to 1.53). Remote outreach was associated with increasing 5-year age (OR1.17, 1.05 to 1.31) and residing in an outer regional/remote location (OR 10.84, 5.82 to 20.19). Specialists based in inner regional areas were less likely than metropolitan-based specialists to provide remote outreach (OR 0.35, 0.17 to 0.70).

**Conclusion:**

There is a healthy level of interest in rural outreach work, but remote outreach is less common. Whilst most providers are metropolitan-based, rural doctors are more likely to provide outreach services. Remote distribution is influenced differently: inner regional specialists are less likely to provide remote services compared with metropolitan specialists. To benefit from outreach services and ensure adequate remote distribution, we need to promote coordinated delivery of services arising from metropolitan and rural locations according to rural and remote health need.

## Background

Outreach is defined as the travel by health workers to provide services away from their normal practice. In 2011, outreach was globally endorsed as an evidence-based strategy to improve universal access to health care in underserved areas [1]. Rural outreach by specialist doctors is supported by Australian policy [2, 3] and research [4] to overcome workforce shortages, address priority areas of care and provide professional support and education for permanent rural health staff. However, it depends on health workers who are willing to travel and their distribution to areas of need. Historically, specialist doctors in Australia have shown a strong interest and investment in outreach work [3], but we lack systematic information about the proportion of specialists that participate in outreach at a national level, the factors predicting participation in outreach work and the provision of outreach in remote areas.

Medical specialist services are relatively centralized in Australia due to their dependence on a viable population base, other staff and technical equipment [5]. Only 15% of specialist doctors, in contrast to 31% of the Australian population, lives in nonmetropolitan (rural and remote) areas [6, 7]. Of specialist doctors based in rural and remote areas, most base their practice in large regional towns (approximate population 50,000 to 100,000) [6]. Remote and outer regional locations have the smallest proportion of medical specialists (constituting 13% and 22% of all doctors compared with 28% and 38% in inner regional and metropolitan areas, respectively) [6].Outside of regional centres, nonmetropolitan Australia is a large country with vast stretches of uninhabited land and a large number of small and dispersed communities, which are located up to 1 000 km from service centres. Remote communities tend to have a higher proportion of indigenous people and widespread poverty. Notably, complex comorbid illness is common, but remote communities have restricted, if any, access to local health care [8, 4]. Despite greater need, remote outreach is challenging due to extreme distances, rugged terrain, more limited infrastructure and lower clinical throughput [9].

To promote specialist redistribution, the Australian government introduced a national specialist outreach policy in 2000 [3], allocated through a competitive tender process. Specialists can gain subsidization for travel, accommodation, equipment lease, time spent up-skilling and travel time for private specialists (or back-filling for salaried specialists) for providing outreach to rural and remote areas designated to have a service need [3]. Whilst the policy is thought to account for only a small proportion of all outreach services [10] it does signal a sustained national commitment to outreach. Information about the predictors of outreach work and remote distribution will inform policy decisions about how to best target the workforce.

Most Australian specialist doctors can participate in outreach work, either through private arrangements, rights to private practice for hospital specialists [11] or as part of hospital employment conditions [12]; but there is a lack of information about how the main practice arrangements influence outreach work participation. Private practice is quite common among specialist doctors in Australia: only 33% of specialist doctors work solely in the public sector whereas 19% solely in the private sector, and 48% are in mixed sector practice, of which 58% work mainly privately in hospital or consulting rooms or both [11].

Different types of specialty doctors have the capacity to practice intermittently in small populations [5], but no single analysis has observed how outreach work varies by specialty. Participation by different specialties may be related to rural health need or formal service plans that designate the services needed. Although there is limited information to assess this, core specialty outreach services routinely needed in remote Australian locations have been proposed [13], and rural health strategy in Australia highlights the importance of generalist specialists (for example, general medicine and general surgery) because of their wide scope of practice [14]. Variation by specialty may also be related to labour market conditions. In the Australian context, specialist doctors might be more likely to practice rural outreach work if competition for their specialty services in metropolitan areas is high, for example, when there is an oversupply of a particular specialist type in metropolitan centres. Specialties experiencing workforce shortages, on the other hand, may have less capacity to provide outreach and could experience weaker market influences driving their participation. A number of specialties have been formally assessed at a national level as experiencing current workforce shortages, including psychiatry, medical oncology, general medicine, paediatric surgery and radiation oncology [15].

This paper is the first national study of rural outreach participation by specialist doctors. It aims to outline the proportion of Australian specialist doctors who participate in rural outreach, describe the characteristics of specialists who provide outreach and assess how these characteristics influence remote distribution. Further, it describes the extent of outreach and its remote distribution by specialist type to discuss some of the broad factors influencing participation by specialty.

## Methods

We used data from the Medicine in Australia: Balancing Employment and Life (MABEL) study. It is a large prospective cohort study that conducts annual waves of data collection using a national database of all Australian doctors (https://mabel.org.au/). The first wave was a census of the Australian medical workforce able to be contacted and working clinically (n = 54,750) between June and November 2008 [16]. Contact details were obtained from the Australian Medical Publishing Company, considered the most comprehensive and accurate listing of all national medical practitioners at the time. Doctors (general practitioners, specialists, specialists in training and hospital non-specialists) were sent an invitation and study information, a paper copy of the survey and were given the opportunity to complete the survey online through a secure website. Three reminders were issued. The survey collected information about job satisfaction, attitudes toward work, work setting, workload, finances, geographic location, demographics and family circumstances [17]. The section on geographic location included questions about the main place of work, main place of residence, and years and location of any childhood rural background. Specialist doctors were also asked whether they travel to provide services/clinics in other geographic areas and were able to list up to three locations (town name and postcode without designating metropolitan or nonmetropolitan locations). A total of 10,498 doctors responded (overall response rate was 19%). Response bias for the wave 1 sample was reported in a previous study using key covariates of age, sex, geographic location, doctor type and hours worked [16]. It was found to be negligible with the potential to adjust minor bias through weighted analysis.

The primary outcome of this paper, outreach participation, was defined as medical specialist doctors travelling to provide clinics/services in at least one nonmetropolitan location. The secondary outcome, remote outreach participation, was defined as a subset of outreach, where services were provided in at least one remote location. Specialist residential and outreach locations were coded using the 5-level Australian Standard Geographical Classification - Remoteness Area scale [18], which is based on the average road distance to nearby larger service centres. The geographic properties of this scale are outlined in Table 1. Nonmetropolitan locations included those categorized as inner regional, outer regional, remote or very remote (an index >1), and remote locations included those categorized as remote or very remote (an index 4 to 5). Specialists who did not report the specific rural town/s they travelled to (for example, reported a broad geographic catchment) were excluded from all analyses because we considered this practice aligned with locum rather than outreach work, which comparatively involves a strong awareness of revisiting specific communities over time.

**Table 1:** **Geographic properties of the Australian Standard Geographical Classification - Remoteness Area scale (ASGC-RA)** [7, 18]

ASGC-RA index	Label	Australia’s population (%)	Australia’s area %	Density (persons per km ^2^)
**1**	Major city	68.6%	0.3%	780
**2**	Inner regional	19.7%	3.8%	16.2
**3**	Outer regional	9.4%	12.5%	2.4
**4**	Remote	1.5%	14.6%	0.3
**5**	Very remote	0.8%	68.7%	0.04

Specialist residential location was re-categorized to three levels: metropolitan (index = 1), inner regional (index = 2) and outer regional/remote (index 3 to 5).

Specialist doctors were those who had completed advanced training in a technical area of care to gain accreditation with a Specialist Medical College. Main specialty was self-reported from a list of 48 accredited specialties that belonged to one of four main specialist groups (Table 2) [17]. Specialists working in a specialty that was not accredited at the time were able to self-report ‘other specialty - not specified above’.

**Table 2:** **Self-reported specialist doctor groups and specialty type,** Medicine in Australia: Balancing Employment and Life **(MABEL) survey**

Specialist group	Specialist type
Internal medicine	Cardiology, clinical genetics, clinical haematology, clinical immunology (including allergy), clinical pharmacology, endocrinology, gastroenterology, general medicine, geriatrics, infectious diseases, intensive care-internal medicine, medical oncology, neurology, nuclear medicine, paediatric medicine, renal medicine, rheumatology and thoracic medicine
Pathology	General pathology, anatomical pathology, clinical chemistry, cytopathology, forensic pathology, haematology, immunology and microbiology
Surgery	General surgery, cardiothoracic surgery, orthopaedic surgery, otolaryngology, paediatric surgery, plastic/reconstructive surgery, urology, neurosurgery and vascular surgery
Other	Anaesthesia (non ICU), dermatology, diagnostic radiology, emergency medicine, intensive care - anaesthesia, medical administration, obstetrics and gynaecology, occupational medicine, ophthalmology, psychiatry, public health medicine, radiation oncology and rehabilitation medicine

Source: MABEL Wave 1 questionnaire [17].

Age quintiles followed a linear distribution with outreach; thus, age was included as a continuous variable grouped in 5-year increments to aid interpretation.

Rural background was defined as the number of childhood years residing in a rural area up until school-leaving age (0 to 18 years) and was categorized into three groups: 0, 1 to 10 and 11 to 18 years.

Private practice was self-reported with three response options: no private practice work (public only), private practice work in a hospital only or private practice in hospital and private consulting rooms.

Cross-sectional sampling weights were applied to all analyses. To determine the predictors of the primary outcome, outreach, bivariate associations of seven covariates (sex, age, location of residence, years of childhood rural background, practice arrangements and specialist group) were tested using logistic regression, odds ratios (OR) and 95% confidence intervals. In this analysis, four specialist groups were used, comprising individual specialist types as outlined in Table 2. Interactions were tested in adjusted models using the Wald test. A single multiple logistic regression model included all these covariates.

To determine the predictors of the secondary outcome, remote outreach, separate logistic regression analysis was done, which included the same covariates as for the primary outcome. This analysis compared specialists who provided remote outreach with those who provided outreach to other rural areas.

The t-test was used to compare mean age between specialists providing outreach or not and to compare those providing remote versus other rural outreach.

Further, separate univariate logistic regression analysis was undertaken to test the association between specialist types and the primary and secondary outcomes. Twelve specialties consisting of all eight pathology specialties (Table 2) and four internal medicine specialties (clinical genetics, clinical haematology, clinical immunology and clinical pharmacology) were grouped together as ‘laboratory-based’ because of their strong links with work in that setting. They were used as the reference group because laboratories tend to be centralized. Medical administration and public health specialties were combined into one group, as were cardiothoracic surgery and neurosurgery, due to small cell sizes. For analysis of remote outreach, the same laboratory-based specialty group was used as the reference group. Specialty types individually analysed were general medicine, paediatric medicine, general surgery, orthopaedic surgery, otolaryngology, anaesthesia (non-ICU), dermatology, diagnostic radiology, obstetrics and gynaecology, ophthalmology, psychiatry and other specialties not specified. Remaining specialist types were combined and included as one group due to small cell sizes.

The study was approved by the University of Melbourne, Faculty of Business and Economics Human Ethics Advisory Group (Ref. 0709559) and the Monash University Standing Committee on Ethics in Research Involving Humans (Ref. CF07/1102 - 2007000291).

## Results

Responses were received in Wave 1 from 4,596 specialists (22% of all specialist doctors). Of these, n = 35 were excluded as they did not list the specific rural location/s they visited. In total, n = 909 (19%) provided rural outreach, of which n = 149 (16%) provided remote outreach. Of rural outreach providers, n = 715 (83%) were male, n = 623 (74%) were metropolitan-based, n = 618 had no years of childhood rural background (70%) and n = 525 (61%) worked in private consulting rooms. Of those providing remote outreach, n = 112 (80%) were male, n = 82 (66%) were metropolitan-based, n = 103 (75%) had no years of childhood rural background and n = 72 (54%) worked in private consulting rooms.

Table 3 shows the characteristics associated with outreach provision.

**Table 3:** **Strength of association between characteristics of Australian specialist doctors and providing rural outreach services**

Covariates of interest	Providing outreach		Univariate odds ratio (95% confidence interval) ^a^	***P***value	Multivariate odds ratio (95% confidence interval) ^a^ ^c^	***P***value
	Yes (n)	% yes ^a^				
**Total**	909	19				
**Sex**						
Female	194	15	Reference		Reference	
Male	715	20	1.47 (1.22 to 1.76)	<0.0001	1.38 (1.12 to 1.69)	0.002
**Age** **(grouped by 5 years)**	909	Mean 50.9 SD 9.68	1.00 (0.97 to 1.04)	0.06	0.98 (0.94 to 1.01)	0.28
**Location of residence** ^**b**^						
Metro	616	17	Reference		Reference	
Inner regional	208	33	2.28 (1.88 to 2.77)	<0.0001	2.07 (1.68 to 2.54)	<0.0001
Outer regional/remote	78	43	3.55 (2.54 to 4.96)	<0.0001	3.40 (2.38 to 4.87)	<0.0001
**Years of Rural background** ^**b**^						
Nil	618	19	Reference		Reference	
1 to 10	103	22	1.23 (0.96 to 1.58)	0.10	1.21 (0.93 to 1.56)	0.16
11+	174	25	1.39 (1.14 to 1.70)	0.001	1.24 (1.00 to 1.55)	0.06
**Practice arrangements** ^**b**^						
Public only	198	18	Reference		Reference	
Private to hospital and consulting rooms	525	22	1.29 (1.06 to 1.56)	0.01	1.24 (1.01 to 1.53)	0.04
Private to hospital only	135	15	0.78 (0.60 to 1.00)	0.05	0.78 (0.60 to 1.02)	0.07
**Specialist group** ^**b**^						
Pathologist	33	16	Reference		Reference	
Surgeon	146	24	1.58 (1.03 to 2.44)	0.04	1.34 (0.82 to 2.19)	0.24
Internal physician	301	24	1.57 (1.04 to 2.37)	0.03	1.57 (0.99 to 2.49)	0.06
Other specialist	425	18	1.13 (0.75 to 1.68)	0.50	1.14 (0.73 to 1.80)	0.56

^a^analysis includes cross-sectional sampling weight.

^b^ *location of residence* missing for n = 7 specialists providing outreach and n = 307 not providing outreach.

*years rural background* missing n = 14 specialists providing outreach and n = 359 not providing outreach.

*practice arrangements* missing n = 51 specialists providing outreach and n = 311 not providing outreach.

*specialist group* missing n = 4 specialists providing outreach and n = 357 not providing outreach. Specialist group included specialist types as outlined in Table 1.

^c^Total missing from multivariate model n = 725.

In the fully adjusted multivariate model, outreach was associated with being male (OR 1.38, 1.12 to 1.69), residing in a rural area (inner regional: OR 2.07, 1.68 to 2.54; outer regional/remote: OR 3.40, 2.38 to 4.87) and working in private consulting rooms (OR 1.24, 1.01 to 1.53). No significant associations were found for age, specialist group or rural background. No effect modification was evident.

Table 4 shows participation in outreach by specialist type. Compared with laboratory-related specialties, general medicine (OR 1.82, 1.06 to 311), renal medicine (OR 3.26, 1.74 to 6.12), otolaryngology (OR 2.21, 1.13 to 4.34), urology (OR 3.63, 1.72 to 7.67), ophthalmology (OR 1.92, 1.17 to 3.14) and radiation oncology (OR 2.68, 1.34 to 5.33) specialties were more likely to provide outreach. Anaesthetists were less likely than laboratory-based specialties to provide outreach (OR 0.56, 0.37 to 0.84).

**Table 4:** **Strength of association between different types of specialty doctors and providing rural outreach services in Australia**

Specialist type	Providing outreach		Univariate odds ratio (95% confidence interval) ^b^	***P***value
	Yes (n) ^a^	% yes ^b^		
Lab-based specialties	51	18	Reference	
Anaesthesia (non-ICU)	68	11	0.56 (0.37 to 0.84)	0.006
Cardiology	20	26	1.63 (0.87 to 3.03)	0.13
Cardiothoracic surgery/neurosurgery	5	12	0.60 (0.22 to 1.63)	0.32
Dermatology	14	24	1.43 (0.70 to 2.92)	0.33
Diagnostic radiology	56	25	1.55 (0.99 to 2.42)	0.06
Emergency medicine	24	13	0.67 (0.38 to 1.15)	0.15
Endocrinology	11	14	0.74 (0.36 to 1.54)	0.42
Gastroenterology	14	17	0.92 (0.47 to 1.80)	0.81
General medicine	32	28	1.82 (1.06 to 3.11)	0.03
General surgery	51	23	1.33 (0.84 to 2.10)	0.22
Geriatrics	24	28	1.82 (1.00 to 3.27)	0.05
Infectious diseases	7	20	1.13 (0.44 to 2.89)	0.8
Intensive care	9	13	0.69 (0.31 to 1.53)	0.37
Intensive care – anaesthesia	4	13	0.69 (0.23 to 2.09)	0.51
Medical oncology	16	30	1.97 (1.00 to 3.86)	0.05
Neurology	15	28	1.75 (0.87 to 3.53)	0.12
Nuclear medicine	5	15	0.82 (0.30 to 2.27)	0.71
Obstetrics and gynaecology	51	19	1.06 (0.68 to 1.67)	0.79
Occupational medicine	14	27	1.72 (0.84 to 3.51)	0.14
Ophthalmology	41	29	1.92 (1.17 to 3.14)	0.01
Orthopaedic surgery	36	23	1.33 (0.81 to 2.19)	0.26
Other specialty	46	23	1.40 (0.87 to 2.23)	0.16
Otolaryngology	17	33	2.21 (1.13 to 4.34)	0.02
Paediatric medicine	68	25	1.56 (1.00 to 2.39)	0.05
Paediatric surgery	5	24	1.44 (0.48 to 4.32)	0.51
Plastic/reconstructive surgery	5	13	0.66 (0.24 to 1.80)	0.42
Psychiatry	82	19	1.04 (0.70 to 1.57)	0.84
Public health medicine/medical administration	6	22	1.32 (0.47 to 3.69)	0.6
Radiation oncology	17	37	2.68 (1.34 to 5.33)	0.005
Rehabilitation medicine	13	23	1.41 (0.69 to 2.86)	0.35
Renal medicine	26	42	3.26 (1.74 to 6.12)	<0.0001
Rheumatology	11	23	1.34 (0.63 to 2.85)	0.45
Thoracic medicine	12	16	0.88 (0.43 to 1.80)	0.73
Urology	15	44	3.63 (1.72 to 7.67)	0.001
Vascular surgery	11	31	2.01 (0.89 to 4.54)	0.09

^a^ *specialist type* missing for n = 7.

^b^analysis includes cross-sectional sampling weight.

Table 5 shows the characteristics influencing remote outreach, compared with other rural outreach. Increasing age (OR 1.17, 1.05 to 1.31) and residing in an outer regional/remote area (OR 10.84, 5.82 to 20.19) were significantly associated with providing outreach in remote locations. Specialists in inner regional areas were less likely to provide remote outreach (OR 0.35, 0.17 to 0.70). Specialists working in private consulting rooms tended to be less likely to provide remote outreach, but this was not significant. Sex, rural background and specialist group were not associated with remote provision. No effect modification was evident.

**Table 5:** **Strength of association between characteristics of Australian specialist doctors and providing remote outreach services, compared with any rural outreach**

Covariates of interest	Providing remote outreach		Univariate odds ratio (95% confidence interval) ^a^	***P***value	Multivariate odds ratio (95% confidence interval) ^ac^	***P***value
	Yes (n)	% yes ^a^				
**Total**	149	16				
**Sex**						
Female	37	18	Reference		Reference	
Male	112	15	0.79 (0.52, 1.19)	0.26	0.75 (0.45 to 1.25)	0.27
**Age** **(grouped by 5 years)**	149	Mean 51.8 SD 10.13	1.06 (0.97 to 1.16)	0.18	1.17 (1.05 to 1.31)	0.006
**Location of residence** ^**b**^						
Metro	82	14	Reference		Reference	
Inner regional	11	5	0.36 (0.19 to 0.70)	0.002	0.35 (0.17 to 0.70)	0.003
Outer regional/remote	54	62	14.65 (8.59 to 25.00)	<0.0001	10.84 (5.82 to 20.19)	<0.0001
**Years of Rural background** ^**b**^						
Nil	103	17	Reference		Reference	
1 to 10	16	13	0.92 (0.52 to 1.63)	0.77	0.83 (0.42 to 1.65)	0.59
11+	25	12	0.84 (0.52 to 1.35)	0.47	0.68 (0.37 to 1.25)	0.22
**Practice arrangements** ^**b**^						
Public only	48	21	Reference		Reference	
Private - hospital and consulting rooms	72	14	0.50 (0.33 to 0.75)	0.001	0.64 (0.39 to 1.06)	0.08
Private - hospital only	24	15	0.68 (0.39 to 1.17)	0.16	0.65 (0.33 to 1.28)	0.21
**Specialist group** ^**b**^						
Pathologist	5	15	Reference		Reference	
Surgeon	45	20	1.38 (0.49 to 3.90)	0.54	1.86 (0.58 to 5.95)	0.30
Internal physician	29	13	0.98 (0.36 to 2.68)	0.98	1.05 (0.34 to 3.18)	0.94
Other specialist	70	16	1.10 (0.41 to 2.96)	0.84	1.46 (0.49 to 4.37)	0.50

^a^analysis includes cross-sectional sampling weight.

^b^ *location of residence* missing n = 2 for specialists providing remote outreach and n = 5 for those providing other rural outreach.

*years rural background* missing n = 5 for specialists providing remote outreach and n = 9 for those providing other rural outreach.

*practice arrangements* missing n = 5 for specialists providing remote outreach and n = 46 for those providing other rural outreach.

*specialist group* missing n = 0 for specialists providing remote outreach and n = 4 for those providing other rural outreach. Specialist group included specialist types as outlined in Table 1.

^c^Total missing from multivariate model n = 69.

Specialists in general medicine (OR 4.45, 1.30 to 15.15, *P* = 0.017), general surgery (OR 3.89, 1.25 to 12.07, *P* = 0.02), otolaryngology (OR 6.25, 1.57 to 8.26, *P* = 0.009) and dermatology (OR 6.62, 1.53 to 28.68, *P* = 0.012) were more likely to provide remote outreach than laboratory-based specialities. Remote outreach by ophthalmology approached significance (OR 2.99, 0.89 to 10.05, *P* = 0.08).

## Discussion

This study provides the first national perspective of the extent and characteristics of rural outreach by the specialist doctors. Providing rural outreach services is relatively common, suggesting there are both unmet needs/demands for services and a healthy level of workforce interest. A smaller proportion of specialist doctors provide outreach in remote areas and this is influenced differently. Factors influencing outreach participation, and then remote distribution, are discussed.

Outreach participation was more common among male specialists; 83% of providers were male. Whilst most Australian specialists (77%) are male, an increasing proportion of qualified specialists and particularly specialists-in-training are female [19]. Female specialists-in-training increased 4.6% between 2008 and 2012 [6]. The gender-related influences on the uptake of outreach work will be important to explore, as will the potential to influence uptake by exposure to outreach work during medical training.

Around three-quarters of outreach providers are metropolitan-based, largely because 85% of Australian specialists reside in metropolitan areas. However, residing in a rural area was strongly related to providing rural outreach. In rural locations, providing services in nearby towns may be relatively convenient, undertaken as part of employment or organizational expectations [12] (under hub-and-spoke regional health models) or provided to increase the viability of regional specialist practice [20]. Furthermore specialists residing in rural locations may be more aware of regional health needs in neighbouring towns [21].

Specialists working in private consulting rooms are more likely to provide outreach, possibly due to higher practice autonomy. With more control over work choice, individual motivations can play out [12] such as a desire to respond to identified health needs, to expand and diversify the main practice base [12], or provide complex health care in challenging situations [22]. Of the 19% of specialist doctors who work in private only practices, 71% have private consulting rooms and of the 48% who work in mixed public/private practice, 36% work mainly in private consulting rooms [11].

Despite evidence that rural upbringing can influence the attraction and retention of rural specialist medical staff [23], having a rural background is not significantly associated with outreach. Specialists with a metropolitan upbringing, may value the professional diversity and challenge of intermittent rural practice [22]. It is likely that the dynamics influencing outreach work vary from those influencing permanent rural appointment.

Our study has additionally highlighted key factors influencing the provision of outreach in remote areas. Whilst only 16% of outreach services were provided in remote areas, only 7% of Australia’s nonmetropolitan population resides in remote locations [7]. Nevertheless, remote service provision is still likely to be under-represented because remote communities are spread across 83.3% of Australia’s land mass, remote health needs are greater than those in rural areas and local specialist services are more commonly not available. Further, the frequency of outreach visiting in remote areas is potentially much lower than in rural areas [4].

Remote outreach is associated with increasing age, suggesting that as professional stability and financial security increases, specialists may be more likely to participate.

Inner regional specialists are the least likely to provide remote outreach, possibly related to the high demand for health care coupled with staff shortages in regional settings [21]. Key logistical barriers may be the time requirements for remote travel [12] and limited efficient travel options to transport them to remote areas (that is, reliance on car travel or the need to travel via major cities to access remote-area flights). Further, they may be more likely to identify, within the region, communities that are in need, precluding the need to travel long-distances to fulfil motivations to support underserved populations [12].

Remote outreach tends to be provided by specialists residing either close to or in remote areas or by metropolitan specialists (two-thirds of all remote providers). This suggests that flexible models of service delivery (rather than integrated hub-and-spoke models) currently underpin remote outreach provision [12]. Flexible models may help capitalize on the specialists willing to travel and enable remote areas to tap into metropolitan supply networks. However, where services arise from varied origins, strong local planning processes are paramount to ensure they complement each other, are well integrated with local community-based services and align with local needs and priorities.

Whilst not statistically significant, specialists with private consulting rooms tend to be less likely to provide remote outreach compared with public-only specialists. Private specialists, remunerated through fee-for-service arrangements, may be deterred by lower clinical throughput [9] and loss of income incurred for the longer travel [12] associated with remote outreach practice. Current Australian outreach policy aims to overcome disincentives for private specialists to travel and up-skill local staff, but not all specialist types are eligible for policy support and the funding is limited [3]. Further, the policy does not permit private specialists to nominate to receive a sessional or salaried payment for clinical services rendered during outreach, except in exceptional circumstances [2].

It is common for different specialist types to provide outreach services, some well above the average participation rate of 19%. However the extent of outreach and its distribution to remote areas varies by specialty. There were some discernable patterns in participation according to needs or service plans in the Australian setting. Consistent with rural health strategy [14], generalist specialists are more likely to provide both rural and remote outreach. Additionally, three of seven core outreach services considered needed in remote areas [13] are associated with remote outreach (general medicine, general surgery and otolaryngology), two with rural rather than remote provision, (paediatrics and ophthalmology) and two had no association with rural or remote outreach (cardiology and obstetrics and gynaecology). This suggests health service planning is potentially important to mobilizing specific specialty types. Ideally, this planning is based on systematic assessment of rural and remote health need and existing workforce capacity, at a regional level.

Workforce shortages in some specialties are unrelated to participation in outreach work. For example, general medicine, radiation oncology and psychiatry are considered to be in current workforce shortage [15], but only psychiatrists are less likely to provide outreach services. Addressing the greater relative health need in rural and remote areas could potentially be a stronger driver than market forces. Motivations that affect different types of specialists require further exploration, but evidence to date shows outreach work is not necessarily a profitable undertaking [3].

In addition to, or instead of, outreach services, rural and remote populations may access specialist medical care through a range of other service models and arrangements. These include: 1) role-substitution with general practitioner proceduralists (for example, anaesthetics); 2) telemedicine (for example, psychiatry); 3) aero-medical retrieval (for example, emergency medicine); 4) patient assisted transport or 5) travel (at patient’s own expense) to regional or metropolitan centres. Decisions about how to deliver services might be influenced by the preferences of local rural staff and patients, the complexity of care with respect to local infrastructure and rural workforce capacity, how practical and affordable it is for patients to travel and the cost and availability of patient and specialist transport and accommodation. Compared with the alternatives, outreach has the potential to provide up-skilling on site, support complex case management [24], enable simple procedures [20], improve continuity of care [4] and reach populations who are unlikely to otherwise seek care [4], but it may not be timely enough in urgent situations, nor have the capacity to reach all communities in need. The way outreach services are billed is yet to be determined systematically, but economies of scale are expressed where the specialist travels to a group of patients, rather than individual patients to the specialist [20].

This paper reflects patterns of outreach in a nation where specialist doctors have a strong history of self-initiating rural outreach work, supported by a universal health insurance system and since 2000, a national outreach policy which helps subsidize travel and accommodation for selected specialists [3]. Also, among Australian doctors, specialists are the highest income earners [25], possible enabling them to provide outreach services in situations of no or limited financial benefit [3]. Depending on the state of the population’s health and locally available workforce and infrastructure in underserved areas, other nations may decide to mobilize primary, preventative or specialty workforces. Factors influencing the participation by other health workers are likely to vary. But for the workforce of interest, it is important to consider the history of outreach practice, public/private work sector balance, workforce size and current distribution, remuneration arrangements, geographical distances and the availability of expedient travel options. In many countries, distances to rural and remote areas may be smaller than in Australia but limited infrastructure, and safety issues may affect mobilisation of the workforce and their travel to remote locations.

Some limitations are acknowledged. This paper was not able to explore the full extent of rural outreach work because the MABEL survey did not collect information about how frequently outreach services were provided to different communities, nor the exact functions the specialist doctors performed at the rural locations they visited. This has potential implications for the type of rural health service capacity achieved by workforce mobilization patterns studied in this paper. The rate of visiting to remote areas is expected to be considerably lower than that to regional areas, but this has not been systematically studied. The MABEL survey is currently collecting information about the frequency of visiting, remuneration arrangements and workforce motivations to address information gaps.

As another limitation, this paper has only examined outreach by specialist doctors, assuming they are visiting in isolation. However, it is possible that participation in outreach is influenced by the ability to visit as part of a complementary health-care team, which helps overcome barriers such as local staff shortages. Whilst we have not analysed outreach with respect to local-level service capacity, we recognize that sustainable outreach is conditional on stable primary health care [26]. Policies to support recruitment and retention of primary health staff (medical, nursing, allied health, indigenous health workers and community volunteers) are likely to improve the distribution of specialist outreach workers, particularly to remote areas. Apart from excluding 35 specialists who did not report the specific locations they visited, this study was not able to further delineate locum from outreach workers due to a lack of information about regularity and length of rural visiting. We tested the effect of excluding specialists who work as hospital locums for more than 15 hours per week in their normal practice (n = 34 providing any rural outreach), which did not affect the results. We used cross-sectional data, such that associations, rather than causal relationships could be examined. A cross-sectional snapshot can be informative because evidence shows outreach tends to be sustained for at least five years [12].

We acknowledge that our findings are based on 2008 data, and we are aware that the policy environment has changed somewhat since that time [3]. We expect that many of the predictive factors studied (age, location, specialist type and practice arrangements) have remained relatively stable over time. Our paper provides the first national study of participation in outreach, providing a baseline from which to examine trends over time. We are pursuing further research on longitudinal trends in the context of changes in policy since 2008.

Whilst we used survey weights to control for selection bias, unobserved response bias that cannot be corrected by the use of weights may exist.

## Conclusions

Outreach is relatively common among Australian specialist doctors suggesting there is a need or demand for services and a healthy level of workforce interest. Whilst rural specialists are more likely to provide outreach, metropolitan areas form an important hub, being the main locations where specialists reside. Despite greater need, remote outreach is less prevalent and depends on harnessing specialists based proximally as well as those from metropolitan areas. Whilst having private consulting rooms may influence outreach work, private specialists may be less inclined to provide remote outreach. Coordinated planning to promote outreach by specific specialties, and integrate services arising from different locations, is important to harnessing the benefit of outreach, specific to community need.

## Abbreviations

MABEL: Medicine in Australia: Balancing Employment and Life
OR: odds ratio.
